# Clonal Diversity of Extraintestinal Pathogenic *Escherichia coli* Strains Isolated from Canine Urinary Tract Infections in Brazil

**DOI:** 10.3390/antibiotics14080819

**Published:** 2025-08-10

**Authors:** Luciana Sartori, João Pedro Rueda Furlan, Fábio Parra Sellera, Fernanda Borges Barbosa, Yohanna Carvalho dos Santos Aoun Chikhani, Gabriel Gandolfi, Terezinha Knöbl

**Affiliations:** 1 Department of Pathology, School of Veterinary Medicine and Animal Science, University of São Paulo, São Paulo 05508-270, Brazil; 2 Paulista School of Medicine, Federal University of Sao Paulo, São Paulo 04039-032, Brazil; 3 School of Veterinary Medicine, Metropolitan University of Santos, Santos 11045-002, Brazil; fsellera@usp.br; 4 Department of Internal Medicine, School of Veterinary Medicine and Animal Science, University of São Paulo, São Paulo 05508-270, Brazil; 5 Department of Internal Medicine, Division of Infectious Diseases, Federal University of São Paulo, São Paulo 04023-062, Brazil

**Keywords:** genomic analysis, antimicrobial resistance, virulence, urinary tract infection, small animal medicine

## Abstract

Background/Objectives: Extraintestinal pathogenic *Escherichia coli* (ExPEC) strains, particularly those belonging to phylogenetic group B2, are clinically significant due to their frequent involvement in urinary tract infections (UTIs) and display antimicrobial resistance profiles. While the association of phylogroup B2 *E. coli* with human urinary tract infections is well established, the growing number of reports of ExPEC strains in canine UTIs highlights their clinical relevance in small animal medicine and raises concerns about their potential role in zoonotic transmission. This study investigated the microbiological and genomic features of *E. coli* strains isolated from dogs with UTIs in São Paulo, Brazil. Methods: Between March and May 2023, a total of 60 *E. coli* strains from canine UTIs were screened for antimicrobial susceptibility and phylotyping. Accordingly, four strains (6.6%) were identified as multidrug-resistant (MDR) or belonging to phylogroup B2 and, therefore, were submitted for characterization by whole-genome sequencing. Results: The four *E. coli* strains exhibited diverse antimicrobial resistance profiles, including resistance to third- and fourth-generation cephalosporins and fluoroquinolones. Phylogenetic groups B1, B2, and G, and sequence types (ST) 73, ST224, ST1193, and ST12960 were identified. The resistome included clinically important β-lactam resistance genes, such as *bla*_CTX-M-55_ and *bla*_CMY-2_, as well as mutations in the quinolone-resistance-determining region. Virulence factors associated with ExPEC pathogenesis, including adhesion, iron acquisition, immune evasion, and toxin, were detected. Plasmid sequences were identified as carrying antimicrobial resistance and virulence genes, highlighting the potential for horizontal gene transfer. Conclusions: Our findings underscore the importance of genomic surveillance in companion animals to better understand the epidemiology of ExPEC strains and monitor the spread of MDR strains.

## 1. Introduction

*Escherichia coli* is a host-generalist Gram-negative bacterium that inhabits the gastrointestinal tract of both humans and animals [1]. However, the species comprises diverse pathogenic lineages, including diarrheagenic *E. coli* and extraintestinal pathogenic *E. coli* (ExPEC), which are responsible for intestinal and extraintestinal infections, respectively [2]. The convergence of virulence and antimicrobial resistance (AMR) in ExPEC strains has become a global health threat, as it limits therapeutic options and increases the risk of unfavorable clinical outcomes [3].

In the ExPEC pathotype, strains belonging to the phylogenetic group B2 are clinically important pathogens, as they are frequently identified in urinary tract infections (UTIs) and exhibit multidrug-resistant (MDR) profiles [4,5]. B2 *E. coli* strains commonly carry virulence genes that promote adhesion, immune evasion, and persistence in the urinary tract [6]. Notably, these strains have been recognized as major causative agents of UTIs in humans [7], with similar trends increasingly reported in companion animals [8,9]. However, data on their occurrence and genomic characteristics in canine UTIs remain more limited. This raises concern about the potential bidirectional transmission of MDR ExPEC, particularly given the close contact between humans and their pets [10].

Given the clinical burden of B2 *E. coli* in both human and veterinary medicine, understanding its prevalence, AMR profiles, and genetic traits in companion animals remains essential. Herein, we conducted a microbiological and genomic investigation of MDR and B2 *E. coli* strains isolated from canine UTIs in Brazil.

## 2. Results

### 2.1. Diverse AMR Profiles, Phylogroups, and Sequence Types in Canine UTI Strains

Four strains (6.6%) were identified as MDR or belonged to phylogroup B2 and, therefore, were submitted to genomic characterization (Table 1). The AMR profiles varied among the *E. coli* strains, with strain VPTEC10 resistant to AMP, CFE, CIP, ENR, and STP; strain VPTEC11 to AMP, CFE, CVN, CRO, CPM, and ATM; strain VPTEC38 to AMP, CFE, CIP, and ENR; and strain VPTEC43 exhibiting resistance exclusively to TRI. The MDR strains were resistant to broad-spectrum antimicrobials, including third- and fourth-generation cephalosporins and fluoroquinolones. By using genomic analysis, the phylogenetic groups were confirmed, except that the B2 of the VPTEC11 strain was corrected to G. Furthermore, *E. coli* strains displayed a genetic diversity of four distinct sequence types (ST), such as ST73, ST224, ST1193, and ST12960, with different CH types and serotypes (Table 1).

### 2.2. AMR and Biocide Resistance Determinants

The resistome analysis identified diverse antimicrobial resistance genes (ARGs), highlighting the extended-spectrum β-lactamases gene *bla*_CTX-M-55_ and cephalosporinase gene *bla*_CMY-2_. Other ARGs associated with resistance to β-lactams (*bla*_TEM-1A_), aminoglycosides (*aadA12*), fosfomycin (*fosA3*), lincosamides [*lnu(A)*], and trimethoprim (*dfrA8*) were also detected. Chromosomal mutations in GyrA, ParC, and ParE, which are associated with fluoroquinolone resistance, were identified in VPTEC10 and VPTEC38 strains. In this context, the AMR genotype corroborates the phenotype found. Furthermore, biocide resistance genes were also detected in the VPTEC10 strain, as follows: *sitABCD* (peroxide) in VPTEC11, VPTEC38, and VPTEC43 strains; and *sitABCD* and *formA* (aldehyde) (Table 1).

### 2.3. ExPEC-Related Virulence Factors

The virulence genes analysis revealed factors commonly associated with ExPEC pathogenesis. Most strains harbor genes linked to adhesion (e.g., *csgA*, *fdeC*, and *yehABCD*), iron acquisition (e.g., *chuA*, *iucC*, and *iutA*), and immune evasion (e.g., *iss* and *ompT*), which are critical for survival and colonization in host extraintestinal environments. Notably, VPTEC11, Ec38, and Ec43 strains carry genes *aslA*, *astA*, and *tsh*, which are implicated in epithelial interactions and toxin production. The presence of *sat*, *usp*, and *vat* genes in VPTEC38 and VPTEC43 strains further indicates their potential for causing tissue damage and immune modulation. In addition, the *irp2*-*fyuA* gene cluster, linked to the *Yersinia* high-pathogenicity island, was also detected in these strains (Table 1).

Capsule-related genes (*kpsE* and *kpsMII*) and the *pap* and *foc* fimbrial operons found in the VPTEC43 strain are commonly found in uropathogenic *E. coli*, reinforcing its ExPEC status. In contrast, VPTEC10 exhibits fewer typical ExPEC traits, with enrichment in fimbrial genes, such as *faeA*–*I*, typically associated with enterotoxigenic *E. coli* (ETEC), suggesting a different or hybrid pathotype (Table 1). Therefore, the virulence profiles of VPTEC11, VPTEC38, and VPTEC43 highlight their strong ExPEC potential, characterized by a convergence of genes that promote adhesion, iron uptake, toxin production, and resistance to host defenses.

### 2.4. Plasmid-Mediated Dissemination of AMR and Virulence Genes

*E. coli* strains harbor various plasmid replicons, including IncF, IncI1, IncN, and IncX families (Table 1). The *bla*_CTX-M-55_ and *bla*_CMY-2_ genes were identified on plasmid contigs. In the VPTEC11 strain, a 2763 bp fragment was identified as harboring the *bla*_CTX-M-55_ gene, which was flanked by ΔIS*Ecp1* and *orf477* elements. In the VPTEC38 strain, an IncI1-Iα plasmid fragment with 102,625 bp in length was identified as carrying AMR (*bla*_CMY-2_) and virulence (*cib*) genes. In this case, the *bla*_CMY-2_ gene was associated with IS*Ecp1*, *blc*, and *sugE* elements. In addition, IncI1-Iα and IncFIB(AP001918) plasmid fragments were detected as harboring virulence genes in VPTEC10 (*faeA-I*), and VPTEC11 (*ompT*, *etsC*, and *hlyF*) strains, respectively.

## 3. Discussion

In this study, we report the occurrence and genomic characteristics of ExPEC strains isolated from canine UTI in Brazil, with a particular concern for those belonging to phylogenetic group B2. Our results contribute to growing genomic evidence linking specific *E. coli* lineages to pathogenicity and AMR in companion animals, reinforcing their clinical and epidemiological significance. The use of whole-genome sequencing (WGS) significantly enhances the accuracy of *E. coli* phylogroup determination and allows for comprehensive characterization of AMR, virulence factors, and mobile genetic elements [11]. Phylogroups B2 and G are closely related lineages within the ExPEC pathotype, often associated with extraintestinal infections [8,12].

Similar ExPEC phylogroups, with a predominance of group B and multiple STs, have been increasingly reported in dogs with UTIs across many countries, often exhibiting comparable patterns of AMR and virulence profiles [9,13,14]. In human medicine, particularly in healthcare settings, these same *E. coli* lineages have been frequently associated with extraintestinal infections and MDR profiles, suggesting that companion animals may serve as spillover hosts of MDR human-associated ExPEC pandemic clones [15]. This overlap raises important One Health concerns, as it indicates that dogs may act as reservoirs or recipients of globally disseminated ExPEC clones. Although studies from Brazil remain limited, the detection of these internationally recognized lineages in São Paulo suggests that similar dynamics are occurring locally and underscores the importance of regional genomic surveillance.

Some *E. coli* strains displayed a MDR profile, with particular concern to resistance to extended-spectrum cephalosporins and fluoroquinolones, which have been widely used as first-line choice therapeutic options for canine UTIs, although their empirical use has been discouraged by the International Society for Companion Animal Infectious Diseases [16]. The detection of clinically important β-lactam resistance genes, which confer resistance to third-generation cephalosporins and cephamycins, along with chromosomal mutations linked to quinolone resistance, supports the AMR phenotypic profiles found and highlights the convergence of resistance to β-lactams and quinolones [17]. Of concern, this AMR phenotype has been increasingly reported in *E. coli* strains from UTIs in both humans and dogs [18]. Additionally, the presence of biocide resistance genes may also suggest possible selective pressure exerted by disinfectants, which have been recognized as coselective agents promoting AMR in healthcare settings [19].

ExPEC strains belonged to four distinct STs. The ST73 is a pandemic ExPEC lineage characterized by conserved virulence factors and increasingly associated with UTIs in both humans and dogs [20,21], whereas the ST224 has been globally recognized as a One Health clone, isolated from a broad diversity of colonized or infected hosts, including animals, humans, and the environment [22,23,24,25], and has been associated with MDR patterns and fatal outcomes in pets [23]. The ST1193 is an emergent high-risk fluoroquinolone-resistant ExPEC and uropathogenic clone [26], recently documented carrying the carbapenemase gene *bla*_KPC-2_ [27]. Conversely, there are no reports of ST12960, although there are four poultry Norwegian genomes publicly available in the EnteroBase [28]. While its virulence and plasmid profiles do not substantially differ from other ExPEC strains, making it difficult to infer clinical relevance at this stage, its occurrence in a canine UTI remains noteworthy.

*E. coli* strains carried multiple virulence genes, including those involved in adhesion, iron acquisition, immune evasion, and toxin production. These factors are consistent with the ExPEC pathogenesis and explain the UTIs caused in the dogs studied [29]. Interestingly, an ExPEC strain also presented fimbrial genes typically found in ETEC strains [30]. Accordingly, hybrid pathotypes may emerge in companion animals, reflecting the plasticity of the *E. coli* genome and its ability to acquire and combine virulence traits from different pathotypes. This genetic flexibility can enhance pathogenic potential and complicate the clinical and epidemiological understanding of infections [31,32,33].

The presence of clinically relevant ARGs, along with virulence factors on plasmids in ExPEC strains from dogs, highlights the potential for horizontal gene transfer and rapid dissemination of these traits in clinical and veterinary settings [34]. Although the mobilization potential of these plasmids was not assessed in vitro in this study, their genetic content raises concerns regarding the dissemination of AMR and pathogenicity determinants across bacterial populations. Additional limitations include the relatively small number of *E. coli* strains analyzed and the narrow focus on a single geographic region. In this context, future studies should encompass additional areas and incorporate clinical data to better understand the epidemiology and impact of MDR ExPEC strains in canine UTIs in this country.

## 4. Materials and Methods

### 4.1. Sample Collection, Bacterial Isolation, and Antimicrobial Susceptibility Testing

Between March and May 2023, an investigation into the cause of UTIs in dogs was conducted at a large private clinical laboratory in São Paulo, the most populous city in Brazil. Accordingly, 60 *E. coli* strains, identified by BD Phoenix (BD Diagnostics, Sparks, MD, USA), were obtained using MacConkey agar (Kasvi, Spain) from urine samples of individual dogs. Antimicrobial susceptibility was assessed using BD Phoenix (BD Diagnostics, Sparks, MD, USA) and disk diffusion methods.

The following antimicrobials were tested: aminoglycosides [streptomycin (STP), amikacin (AMK), and gentamicin], β-lactams [amoxicillin–clavulanic acid (AMC), ampicillin (AMP), ampicillin–sulbactam, cephalexin (CFE), cefovecin (CVN), ceftriaxone (CRO), and aztreonam (ATM)], fluoroquinolones [ciprofloxacin (CIP) and enrofloxacin (ENR)], tetracyclines [doxycycline (DOX)], nitrofurans (nitrofurantoin), and sulfonamides (sulfamethoxazole-trimethoprim and trimethoprim). All results were interpreted using the breakpoints available from the Brazilian Committee on Antimicrobial Susceptibility Testing [35], except for AMK, AMC, AMP, CFE, CVN, ENR, and DOX, for which the guidelines of the Clinical and Laboratory Standards Institute [36] were used. Furthermore, MDR was established using the criteria established by Magiorakos et al. [37].

### 4.2. E. coli Phylotyping

Genomic DNA was extracted [38] and conventional polymerase chain reactions were used to determine the phylogenetic groups A, B1, B2, and D, using the genes *chuA* and *yjaA,* and the fragment of TspE4.C2 DNA. The phylogenetic groups were determined according to the following criteria: A [*chuA*^(−)^/TspE_4_.C_2_^(−)^]; B1 [*chuA*^(−)^/TspE4.C2^(+)^]; B2 [*chuA*^(+)^/*yjaA*^(+)^]; and D [*chuA*^(+)^/*yjaA*^(−)^] [39].

### 4.3. Whole-Genome Sequencing and Analysis

Genome sequencing was carried out using the Illumina MiSeq (Illumina, Inc., San Diego, CA, USA) or MinION platforms (Oxford Nanopore Technologies, Oxford, Oxfordshire, UK). The draft genomes were de novo assembled by SPAdes v.3.1.5 or Flye v.2.9.1. Phylogenetic groups were assigned using ClermonTyping v.23.06 (http://clermontyping.iame-research.center/, accessed on 23 April 2025). Multilocus sequence typing, CH typing, serotype, resistome, virulome, and plasmid replicons were determined using MLST v.2.0, CHTyper v.1.0, SerotypeFinder v.2.0, ResFinder v.4.7.2, VirulenceFinder v.2.0, and PlasmidFinder v.2.1, respectively, which are available at the Center For Genomic Epidemiology (https://www.genomicepidemiology.org/, accessed on 23 April 2025). All bioinformatic tools were used with their default parameters. Genetic environments were analyzed using ISfinder (https://www-is.biotoul.fr/index.php, accessed on 23 April 2025) and Nucleotide BLAST (https://blast.ncbi.nlm.nih.gov/Blast.cgi, accessed on 23 April 2025).

## 5. Conclusions

This study provides genomic insights into ExPEC strains associated with canine UTIs in Brazil. The identification of medically important AMR genes and virulence markers commonly found in human clinical settings raises concerns about the potential of these successfully adapted pathogens to persist in companion animal populations and the possible risk of cross-species transmission between pets and their owners. These findings reinforce the importance of continued genomic epidemiological surveillance to better understand the dynamics of ExPEC infections in both veterinary and human medicine.

## Figures and Tables

**Table 1:** Phenotypic and genotypic characteristics of *Escherichia coli* strains isolated from UTIs of dogs in Brazil.

Strain	AMR Profile ^1^	PG ^2^		ST ^3^	CH Type	Serotype ^4^	Resistome ^5^					Virulome	Plasmid Replicon
		PCR	WGS				ARG	Mutation			BRG		
								GyrA	ParC	ParE			
VPTEC10	AMP, CFE, CIP, ENR, STP	B1	B1	224	4–54	O8:H32	*bla*_TEM-1A_, *aadA12*, *lnu(A)*	S83L, D87N	S80I	S458A	*sitABCD*, *formA*	*csgA*, *faeA*, *faeB*, *faeC*, *faeE*, *faeF*, *faeI*, *fdeC*, *gad*, *hlyE*, *hlyF*, *iss*, *lpfA*, *nlpI*, *ompT*, *sitA*, *terC*, *traJ*, *traT*, *yehABCD*	IncFIB(AP001918), IncFIB(pB171), IncFII, IncI1-Iα
VPTEC11	AMP, CFE, CVN, CRO, CPM, ATM	B2	G	12,960	45–222	O149:H34	*bla*_CTX-M-55_, Δ*bla*_TEM-1_, *fosA3*	na	na	na	*sitABCD*	*aslA*, *anr*, *astA*, *cea*, *chuA*, *cma*, *csgA*, *etsC*, *fdeC*, *gad*, *hlyF*, *iroN*, *iss*, *iucC*, *iutA*, *lpfA*, *nlpI*, *ompT*, *sitA*, *terC*, *traJ*, *traT*, *tsh*, *yehABCD*	IncFIB(AP001918), IncFIC(FII), IncFII(pHN7A8), IncN
VPTEC38	AMP, CFE, CIP, ENR	B2	B2	1193	14–64	O75:H5	*bla*_CMY-2_	S83L, D87N	S80I	L416F	*sitABCD*	*aslA*, *chuA*, *cib*, *csgA*, *fdeC*, *fyuA*, *gad*, *iha*, *irp2*, *iucC*, *iutA*, *kpsE*, *nlpI*, *ompT*, *sat*, *sitA*, *terC*, usp, *yfcV*, *yehABCD*	IncI1-ST12, ColpEC648
VPTEC43	TRI	B2	B2	73	24–30	ONT:H1	*dfrA8*	na	na	na	*sitABCD*	*aslA*, *chuA*, *clbB*, *csgA*, *fdeC*, *focCsfaE*, *focG*, *focI*, *fyuA*, *gad*, *hha*, *iroN*, *irp2*, *iss*, *iucC*, *iutA*, *kpsE*, *kpsMII*, *nlpI*, *ompT*, *papA_F7-2*, *papC*, *pic*, *sat*, *terC*, *traJ*, *usp*, *vat*, *yehABCD*	IncFIB(AP001918), IncFII(29), IncX3

^1^ AMP, ampicillin; ATM, aztreonam; CFE, cephalexin; CVN, cefovecin; CRO, ceftriaxone; CIP; ciprofloxacin; ENR, enrofloxacin; STP, streptomycin; TRI, trimethoprim. ^2^ PG, phylogenetic group; PCR, polymerase chain reaction; WGS, whole-genome sequencing. ^3^ ST, sequence type. ^4^ NT, not typeable. ^5^ ARG, antimicrobial resistance gene; BRG, biocide resistance gene; na, not applied.

## Data Availability

The sequenced data are available online at the National Center for Biotechnology Information-NCBI (BioProject number PRJNA1276606).
